# Management of Secondary Angle Closure Glaucoma

**DOI:** 10.5005/jp-journals-10008-1157

**Published:** 2014-01-16

**Authors:** Annadurai Parivadhini, Vijaya Lingam

**Affiliations:** Associate Consultant, Department of Glaucoma, Smt Jadhavbai Nathmal Singhvee Glaucoma Services, Sankara Nethralaya, Chennai, Tamil Nadu India; Director, Department of Glaucoma, Smt Jadhavbai Nathmal Singhvee Glaucoma Services, Sankara Nethralaya, Chennai, Tamil Nadu India

**Keywords:** Pupillary block, Neovascular glaucoma, Iridocorneal endothelial syndrome, Aqueous misdirection syndrome, Ciliary body swelling.

## Abstract

Secondary angle closure glaucomas are a distinct entity from primary angle closure glaucoma (PACG). Unlike PACG, secondary angle closure glaucoma's have an identifable contributory factor/s for angle closure and obstruction of aqueous fow which is usually unrelieved by iridotomy. The treatment of each type of secondary angle closure glaucoma is varied, so identification of the primary cause aids in its effective management.

**How to cite this article:** Annadurai P, Vijaya L. Management of Secondary Angle Closure Glaucoma. J Current Glau Prac 2014;8(1):25-32.

## INTRODUCTION

In secondary angle closure glaucoma, the underlying cause can close the angle directly by local iris and angle factors or by acting to move the crystalline lens forward causing pupillary block (secondary pupillary block). This is important since some of these patients with secondary pupillary block will respond to laser iridotomy. They are common causes of glaucoma and can produce high elevations of intraocular pressure (IOP) and ocular morbidity. This review will discuss the risk factors, signs and symptoms, pathophysiology, imaging and the treatment modalities of secondary angle closure glaucomas.

Secondary angle closure glaucoma's can be categorized by the existence of pupillary block or not though some overlap can be seen (Table 1).

### Aqueous Misdirection Syndrome

The aqueous misdirection syndrome is a form of secondary angle closure glaucoma occurring postsurgery with raised intraocular pressure (IOP), shallow or fat anterior chamber (AC) in the presence of a patent peripheral iridectomy (PI)^1^ (Figs 1A to C). It is unresponsive to miotic or fltering surgery. It can occur after fltering surgery,^2^ cataract^3^/com-bined surgery, surgical peripheral iridectomy, following suturelysis,^4^ glaucoma drainage device implantation^5^ or even after laser iridotomy.^6^ The predisposing factors are preexisting angle closure glaucoma, shallow anterior chamber due to wound leak or overflteration.

The pathophysiology is not completely understood, but is believed that the primary mechanism is a blockage of anterior aqueous fow at the level of the ciliary body combined with an inherent impermeability defect in the anterior hyaloid.^7^ Recently, choroidal expansion has been proposed as a contributory factor as evidenced by ultrasound biomicro-scopy (UBM) studies showing fuid in the suprachoroidal space in some patients with ciliary block glaucomas.^8^

### Clinical Features

It is seen in the postoperative period anytime from the 1st day to weeks, sometimes months later. The features are axial shallowing of AC, high IOP or normal IOP in case of functioning blebs, patent PI, closed angle on gonioscopy and ciliary processes can be seen rotated forward pressing against the base of the iris in case of choroidal effusions. UBM shows anterior rotation of ciliary processes pressing against the lens equator (or anterior hyaloid in aphakia).^9^

The principles of treatment are to relieve the obstruction of aqueous fow and restore normal intraocular pressure by medical therapy, to surgically correct the block to aqueous fow, re-establish a normal aqueous fow pathway and drain the aqueous from its abnormal location.

### Medical Therapy

Rule out pupillary block by verifying or creating a patent iridectomy/iridotomy. Start mydriatic-cycloplegic therapy^10^ consisting of 1% atropine and 5% phenylephrine twice a day in phakic and pseudophakic eyes. This tightens the lens– zonular diaphragm to resist the force from behind and also dilate the ciliary body ring to move the ciliary body away from peripheral anterior hyaloid. Fifty percent success rate has been noted with this therapy. Additionally, topical steroids, aqueous suppressants are used to reduce infa mmation and IOP. Osmotics help to lower the IOP as well as to reduce the vitreous volume. Conservative treatment is advised for 5 days to see for resolution.

YAG hyaloidotomy disrupts the anterior hyaloid face allowing aqueous to drain out of the vitreous. It's an elective procedure done in pseudophakics and aphakics through the surgical iridectomy, first perforating the posterior lens capsule and then the hyaloid face. Slight deepening of the anterior chamber is noticeable immediately, dramatic deepening is noticeable after 12 to 24 hours.

**Fig. 1A:**
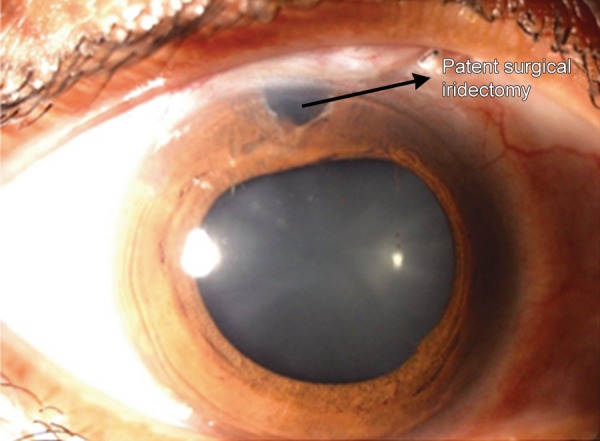
Aqueous misdirection post fltering surgery showing a patent surgical iridectomy

**Fig. 1B:**
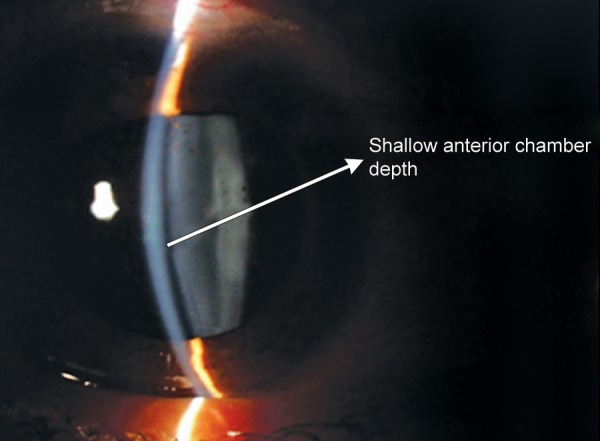
Slit photo showing shallow anterior chamber

**Fig. 1C:**
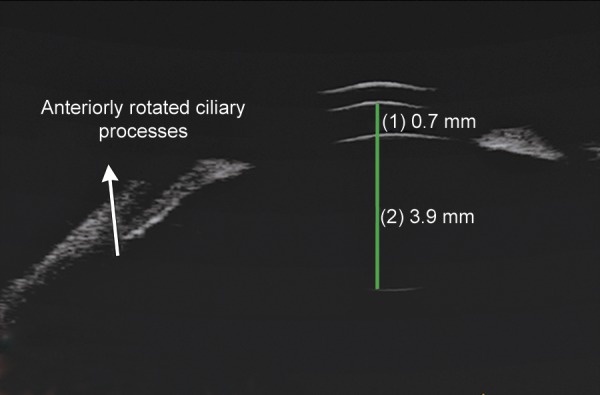
UBM showing shallow anterior chamber with anterior rotation of ciliary processes

**Table 1:** **Table 1:** Classification of secondary angle closure glaucomas

*With pupillary block*		*Without pupillary block*	
Aqueous misdirection		Neovascular glaucoma	
		Iridocorneal endothelial syndromes (ICE)	
		Infammatory glaucoma	
		Ciliary body cysts, tumors	
		Scleral buckling and postvitreoretinal	
		procedures	
		Ciliary body swelling secondary to:	
		a. Central retinal vein occlusion (CRVO)	
		b. Panretinal photocoagulation (PRP)	
		c. Drugs or infammation	

### Surgical Therapy

In cases of medical and laser therapy failure, vitreous surgery almost always cures this condition. The surgery is intended to establish an opening through the anterior hyaloid membrane and the vitreous body for aqueous humour to escape forward to the posterior and anterior chambers. Pars plana vitrectomy^2,3^ is done in pseudophakic and aphakic eyes and IOP control is achieved in 67 to 100% of cases. Vitrectomy-phacoemulsificaton-vitrectomy^11^ was studied in 5 phakic eyes, the preliminary vitrectomy was limited to core vitrectomy to debulk the vitreous, the residual vitrectomy, zonulohyaloidectomy and peripheral iridectomy was performed to create a free communication between the anterior and posterior chambers. Cyclodiode laser photocoagulation has also been proposed in patients with failed medical therapy to break the cycle of aqueous misdirection and prevent future recurrences.^12^

YAG PI done preoperatively in occludable eyes can reduce aqueous misdirection. In patients with increased risk like in decompression related shallowing of the anterior chamber, using viscoelastics during surgery, tighter closure and cycloplegics after surgery prevents aqueous misdirection. It must be remembered that recurrence can occur so vigorous follow-up and chronic atropine drops may be necessary. The fellow eye is also at a high-risk for aqueous misdirection.

### Neovascular Glaucoma

Neovascular glaucoma (NVG) arises in response to retinal ischemia, the common predisposing factors being central retinal vein occlusion and diabetic retinopathy. An NVG patient requires a broad diagnostic workup to determine the underlying cause^13^ and also a predisposed patient requires careful monitoring to detect NVG in its earliest stages.

The pathogenesis of NVG is retinal ischemia which produces angiogenic factors (VEGF, angiogenin, PDGF, TGF-α, β, TNF-α) which in turn causes new vessel formation on the iris and angle leading to formation of fibrovascular membrane which eventually contracts to form peripheral anterior synechiae (PAS) and ultimately complete closure of the angle. VEGF levels are elevated to 40 to 100 times normal in aqueous and vitreous of patients with rubeosis and NVG.^14^

The clinicopathologic course and treatment may be described in the following stages.^15^

Rubeosis Stage (Preglaucoma)

Neovascularization is typically first seen in the pupillary border;^16^ however, gonioscopy should be done in all patients under the risk to look for angle neovascularization, since it can precede iris neovascularization.^17^ The IOP is usually normal, rubeosis is seen as fine randomly oriented vessels near pupillary border or may be first seen in the angle in diabetic and CRVO patients.^18^ Pan retinal photocoagulation (PRP) is indicated which decreases the retinal oxygen demand, thereby reducing the stimulus for release of angio genesis factors.^19^

Open-angle Glaucoma Stage

The rubeosis is more forid in this stage with elevated IOP and open angles with intense neovascularization of the angle. PRP can reverse IOP elevation in this stage. Aqueous suppressants, topical corticosteroids and atropine are used.

Angle Closure Glaucoma Stage

PAS formation can eventually close the angle with ectropion uveae formation resulting in severe glaucoma requiring surgical intervention. PRP is still useful in this stage to reduce IOP if synechial closure is less than 270°^20^ and even if the angle is closed for more than 270° PRP is useful in reducing anterior segment neovascularization before any surgical intervention.^21^

Filtering surgery is rarely successful because of the risk of bleeding and postoperative progression of fibrovascular membrane. In a study in NVG patients, mitomycin C (MMC) augmented trabeculectomy yielded success rates of 62.6, 58.2 and 51.7% at 1, 2 and 5 years respectively.^22^ Modifed trabeculectomy can be performed with intraocular bipolar cautery of peripheral iris and ciliary processes to prevent bleeding.^23^ Intracameral Bevacizumab (avastin)^24^ has been tried in NVG patients resulting in rapid regression of the iris and angle neovascularization and could act as an adjunct in the surgical treatment of these patients. Preoperative intravitreal bevacizumab combined with mitomycin C augmented trabeculectomy has been studied to be a safe and effective method for controlling IOP in NVG, though long-term effects are not known.^25^ Even comparative studies between combined intravitreal bevacizumab (IVB) and trabeculectomy with MMC verses trabeculectomy with mitomycin C alone concluded that IVB significantly reduces postoperative hyphema and controls early postoperative IOP with no difference in long-term results.^26^.

GDDS have gained wide acceptance as a primary procedure for NVG patients because the tube bypasses the fibro -vascular membrane. The aqueous tube shunts have reported success rates of 22 to 97% in these patients.^27,28^ Molteno tube shunts^29^Ahmed glaucoma valve^30^ and Baerveldt glaucoma implants^31^ have shown comparable success rates. Successful IOP control was achieved with com bined pars plana vitrectom y and glaucoma drainage implant in selected patients with refractory NVG.^32^

In eyes with little or no visual potential, treatment is mainly with topical cycloplegics, steroids and aqueous sup-pres sants. Ablation of the ciliary body is undertaken in cases of symptomatic patients. Bloom et al reported a mean IOP reduction of 53% in 25 patients with diode laser for refractory glaucoma.^33^ However of note is the study by Ramli et al^34^ that underlying NVG was a significant risk factor for hypotony post transscleral cyclophotocoagulation. A less aggressive energy setting and a more limited treatment was advised to prevent hypotony.

### Iridocorneal Endothelial Syndromes

Iridocorneal endothelial (ICE) syndrome is a spectrum of ocular diseases characterized by corneal endothelial abnormalities, unilateral glaucoma and iris stromal abnor-malities.^35^ They include progressive iris atrophy, Chandler syndrome and Cogan-Reese syndrome (Figs 2A to C). They may be regarded as different manifestations of the same disease process. It is caused by an abnormal corneal endo-thelium which forms a membrane (ICE membrane) over the anterior surface of the iris and the angle structures, which on contraction distorts the iris, forms peripheral anterior synechiae and closes the angle leading to glaucoma.^36^ Half of all the patients of ICE syndrome develop glaucoma.^37^

Management of glaucoma in ICE syndromes is difficult and medical therapy is restricted to aqueous suppressants which is often unsuccessful.^38^ The success rate of fltering surgery is also lower than most other forms of glaucoma.^39,40^ A study of surgical outcome of trabeculectomy with MMC in 10 ICE syndrome patients showed 8 of 10 patients having adequate IOP control after fltering surgery with mitomycin-C, with a mean follow-up of 14.9 months. The results of their study suggested that trabeculectomy with adjunc tive mitomycin-C may offer better intermediate-term success than trabeculectomy alone or trabeculectomy with 5-fluorouracil.^41^ Failure of fltering surgery is often due to the continued growth of the endothelial membrane over the fltration site.

**Fig. 2A:**
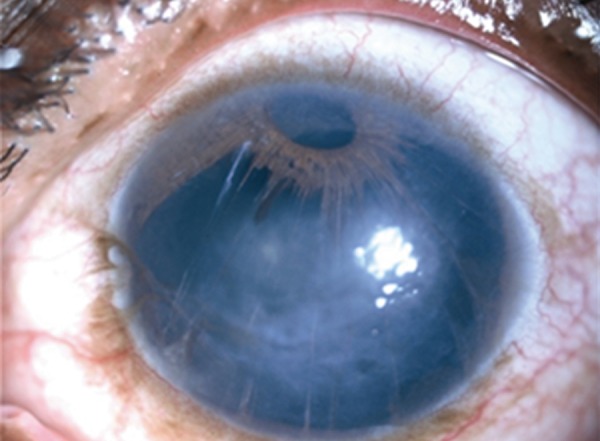
Progressive iris atrophy

**Fig. 2B:**
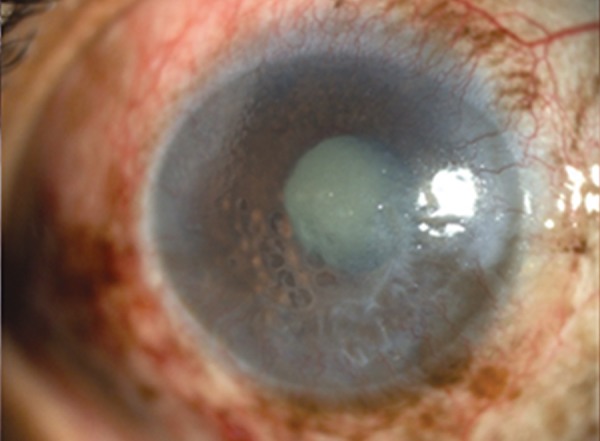
Chandler's syndrome

**Fig. 2C:**
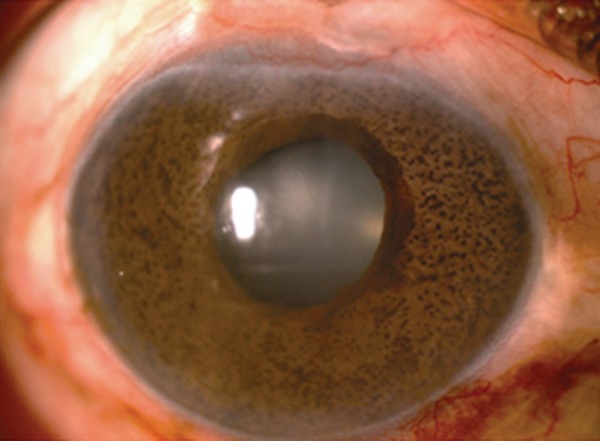
Cogan-Reese syndrome

With GDDs the growth of ICE membrane over the ostium is bypassed. A long-term study of 10 patients with ICE syndrome^42^ showed IOP control of less than 21 mm Hg in four patients and in three required tube repositioning due to membrane formation. They proposed that in ICE patients the tube should be left long in the anterior chamber away from the potential source of ICE cells and entry through pars plana can be used in pseudophakic vitrectomized eyes. Cyclophotocoagulation is indicated in eyes with refractory glaucoma who have failed trabeculectomy or shunt surgery.

### Infammatory Glaucoma

Glaucoma is a potentially devastating complication of uveitis and remains a therapeutic challenge despite availability of new modalities of treatment in both the conditions.^43^

Inflammation can produce secondary angle closure glau coma with pupillary block mechanism due to pos terior synechiae formation or without pupillary block due to infa m matory peripheral iris swelling, exudates in the angle contrac ting to form PAS or due to forward rotation of the ciliary body. PAS form easily in eyes with shallow anterior chamber and in eyes with chronic infammatory processes.

Infammatory glaucoma can occur after trauma, surgery, idiopathic infammatory condition or due to specific uveitic entities.

### Principles of Management

Usually control of infammation alone leads to normalization of IOP. Management of infammation is done by topical or systemic corticosteroids, mydriatics-cycloplegics and systemic immunosuppressives. Medical therapy to control IOP basically involves aqueous suppressants. Intracameral tissue plasminogen activator has been reported to be of use in impending pupillary block glaucoma due to acute fibrin-ous HLA-B27 uveitis.^44^

YAG PI is done for pupillary block glaucomas, although fibrin can close small iridotomies in an infamed eye.^45^ When this is unsuccessful surgical iridectomy is useful if less than 75% of the angle is closed by PAS.^46^

If medical or laser therapy is unable to control the IOP then fltration surgery has to be considered. Trabeculectomy in uveitic eyes generally carries less success due to acceleration of wound healing by postoperative fibrinous and cellular responses.^47^ Antimetabolites like 5FU and Mitomycin C are used to increase the success of trabeculectomy in uveitic eyes (5FU, MMC). A success of 95% (IOP less than 21 mm Hg with 1 or no medication) was achieved with MMC augmented trabeculectomy.^48^ Aggressive anti-infammatory therapy should be given before and after trabeculectomy in these patients.

All the three glaucoma drainage implants (Molteno, Baerveldt and Ahmed valve) have been tried with a success of 79% with Molteno implants,^49^ 94% with Ahmed valve^50,51^ and 91.7% with Baerveldt implant.^52^

Trabeculodialysis^53^ (a modifed goniotomy) was studied in children with infammatory glaucomas with a success of 60%. Goniotomy in chronic childhood uveitis was reported to be successful in 75% of their patients by Freedman et al.^54^

Transscleral diode cyclophotocoagulation is considered in low visual potential eyes; however, the rate of hypotony in uveitis eyes can be higher (19%) than other refractory glaucomas.^55^

### Ciliary Body Cysts

Benign iris/ciliary body cysts can cause glaucoma if they are multiple by pushing the peripheral iris forward to close the angle and cause raised intraocular pressure. The onset of glaucoma can mimic acute angle closure glaucoma. It is also called as pseudoplateau iris since it produces a clinical picture identical to plateau iris. Cysts should be suspected when plateau iris configuration appears more in one segment of the angle giving a characteristic bumpy contour to the peripheral iris. They are often multiple and diffusely distri-buted.^56^ Around 10% of the iris cysts can because glaucoma associated with or without pupillary block glaucoma. They can be confirmed and their extent can be noted by UBM.^57^

Laser iridotomy,^58^ Argon laser iridoplasty,^59^ Laser cystostomy and intermittent pilocarpine therapy^60-62^ have been described as therapeutic approaches.

### Glaucoma following Scleral Buckling Procedures

Here angle closure glaucoma is produced by swelling of the ciliary body due to impaired venous drainage from the vortex veins by the scleral buckle. The incidence of angle closure glaucoma after scleral buckling procedures range from 1.4 to 4.4%.^63^ The risk factors are pre-existing narrow angles, use of an encircling band, placement of the band anterior to the equator and high myopia.

It usually resolves spontaneously over several days to weeks. Cycloplegics are used to shift the lens-iris diaphragm posteriorly by relaxing the ciliary muscle and topical steroids to reduce the infammation. IOP is reduced by means of aqueous suppressants. Miotics should be avoided. Laser PI is not beneficial; rather laser iridoplasty is useful in opening the angle in some of these patients.^64^

GDDs offer an alternative approach in uncontrolled glaucoma as evidenced by the study done by Scott Ingrid U et al with Baerveldt glaucoma implant in eyes with preexisting episcleral bands where IOP control was achieved in all 16 patients with or without medications during the follow-up period of 19.1 to 45.5 months with none of the usual complications reported with glaucoma drainage devices (GDD).^65^

Intraocular gases have been increasingly used in vitreo-retinal surgeries for tamponade effect on the retinal breaks. The gases used are air, Perfluoropropane (C3F8), sulphur hexafluoride (SF6). SF6^66^ expands to twice its volume within 24 to 48 hours and stays in the eye for 10 to 14 days, C3F8 expands to four times its volume in 48 to 72 hours and stays for 55 to 65 days.^67^ The increase in the volume of the gas causes anterior displacement of the lens-iris diaphragm even when prone position is maintained. The IOP rise is high during the period of maximum expansion of gas. The incidence of IOP elevation with SF6 has been reported to range from 6.1 to 67%, maximum with 100% SF6 and minimum with 20% SF6.^68^ C3F8 causes IOP elevation of 18 to 59%, minimum when 14% of C3F8 is used.^69^ Patients should be instructed to maintain head down position to prevent forward movement of iris lens diaphragm. They should be warned against air travel due to IOP variation during atmospheric changes.^70^

Medical therapy consists of aqueous suppressants. If IOP is not responsive to medical therapy then aspiration of a portion of gas may be performed to lower IOP. Laser PI is useful in pupillary block.

### Glaucoma after Silicone Oil Injection

Silicone oil is used as a vitreous substitute for retinal tampo-nade. It can produce glaucoma by pupillary block, infa-mmation, synechial closure, neovascularization, migration of oil into anterior chamber or by open angle mechanism. Secondary glaucoma after silicone oil injection has been reported to be in 6 to 30% of eyes.^71^ Angle closure glaucoma can occur after silicone oil injection due to synechial closure. Overflling of the oil in the eye has to be avoided to prevent secondary glaucoma. Inferior prophylactic iridectomy prevents pupillary block in pseudophakic and aphakic eyes. Medical therapy consists of aqueous suppressants, cortico-steroids and cycloplegics. Postoperative closed iridectomies due to fibrin can be relieved by laser PI.

Silicone oil removal^72^ with or without concurrent glaucoma surgery has been performed to lower IOP, but oil removal carries some risk of retinal detachment. In patients with complete synechial closure silicone oil removal alone cannot be expected to lower IOP, rather glaucoma surgery is indicated in such cases and the decision to remove silicone oil or not depends on the relative risk of redetachment on oil removal and emulsification of oil.^73^ If silicone oil remains in the eye, the GDD should be positioned in the inferior quadrant.^74^

Cyclophotocoagulation by transscleral cytophotoco-agulation (TSC) or endo cytophotocoagulation (ECP) can be used^75^ in refractory cases. Eyes with relatively intact central visual acuity are appropriate candidates for ECP and can be combined with intraocular surgery.^76^

### Ciliary Body Swelling

Secondary angle closure glaucoma can occur due to ciliary body (CB) swelling secondary to vortex vein obstruction (venous stasis), drugs or infammation.

Vortex vein obstruction occurs secondary to extensive PRP or CRVO producing angle closure glaucoma similar to scleral buckling procedures. It is a self limiting condition and will resolve with or without medical therapy. Laser PI has no role in this condition.

**Figs 3A and B:**
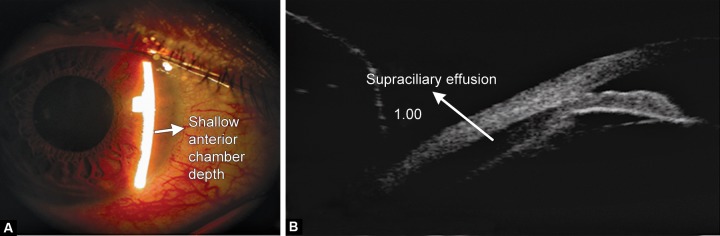
(A) Shallow anterior chamber depth due to topiramate induced angle closure, (B) UBM showing supraciliary effusion following topiramate intake causing secondary angle closure

Ciliary body swelling secondary to drug use is an idiosyncratic reaction causing relaxation of zonules, anterolateral rotation of the ciliary body leading to anterior displacement of iris–lens diaphragm producing induced myopia. Cho-roidal detachment and supraciliary effusions are frequently present detected by UBM. Sulfa-based drugs are associated with this type of secondary angle closure glaucomas like topiramate^77^ (Figs 3A and B), acetazolamide,^78^ hydrochloro-thiazide and contrimoxazole. It presents as bilateral angle closure glaucoma. The management requires stopping of the drug, instituting aqueous suppressants and cycloplegics.^79^ Miotics, topical and systemic carbonic anhydrase inhibitors are contraindicated and laser PI has no role.

## CONCLUSION

Secondary angle closure glaucomas require meticulous history taking, clinical examination, if necessary anterior segment imagings like UBM to identify the specific cause if possible and formulate a treatment plan to avoid ocular morbidity.
